# Draft genome sequence of *Bremerella cremea* LHWP2 isolated from dead ark clam revealing potential nitrogen metabolism pathways

**DOI:** 10.1128/mra.00974-24

**Published:** 2024-12-13

**Authors:** Jin-Hee Seo, Kyung June Yim, Jongbin Lim, Ji-Yeon Chun, Hae-Won Lee

**Affiliations:** 1 Department of Food Bioengineering, Jeju National University, Jeju, South Korea; University of Southern California, Los Angeles, California, USA

**Keywords:** *Bremerella cremea*, LHWP2, ark clam, nitrogen metabolism, Draft genome sequence

## Abstract

We present the draft genome sequence of *Bremerella cremea* LHWP2, a notable member of the Planctomycetes–Verrucomicrobia–Chlamydiae group, isolated from a dead ark clam. The 6,211,343-bp genome contains 5,304 coding gene sequences with a guanine–cytosine content of 56.5 %. The draft genome also reveals potential nitrogen metabolic pathways.

## ANNOUNCEMENT

*Bremerella cremea*, a member of the Planctomycetes–Verrucomicrobia–Chlamydiae group, is an aerobic, budding, motile, and ovoid bacterium within the phylum Planctomycetes (1). This bacterium was originally identified as *Blastopirellula cremea*, but was reclassified as *Bremerella cremea* (1). The rationale for this reclassification stemmed from new insights obtained through comprehensive analyses, including 16S rRNA gene identities, average nucleotide identity, and other molecular markers such as the *rpoB* sequence (2). The bacterium was isolated from a dead ark clam (*Scapharca broughtonii*) found along the southern coast of Korea (1). It was cultured on marine agar medium using the spread plate method and incubated at 30°C for 5 days. The colonies obtained were subcultured three times to ensure purity. Genomic DNA was extracted from multiple colonies obtained from the third subculture.

Genomic DNA of *Bremerella cremea* LHWP2 was extracted and purified using Maxwell Prokaryote/Eukaryote SEV DNA Purification Kit (Promega Corporation, USA) (3). Libraries were generated using the TruSeq Nano DNA kit, and sequencing of the draft genomic DNA was performed. In addition, 151-bp paired-end reads were determined using Illumina NovaSeqX platform (3). A total of 15,133,386 reads spanning 2,285,141,286 bp were generated. The Q30 value was 94.6%. Shovill Galaxy version (v1.1.0) (4) was used for sequence reads, which were trimmed and assembled by Trimmomatic (v0.39) (5) and SPAdes (v3.14.3) (6) in Shovill Galaxy Version. Default parameters were used for all software unless otherwise specified. Trimmed and assembled sequence reads resulted in 51 contigs totaling 6,211,343 bp, which were annotated by RAST (Rapid Annotation using Subsystem Technology) pipeline (7). However, the National Center for Biotechnology Information PGAP (Prokaryotic Genome Annotation Pipeline) (8) processed the genome into 16 contigs by removing contigs shorter than 200 bp, with an estimated coverage of 368-fold as calculated by Shovill.

**Fig 1:**
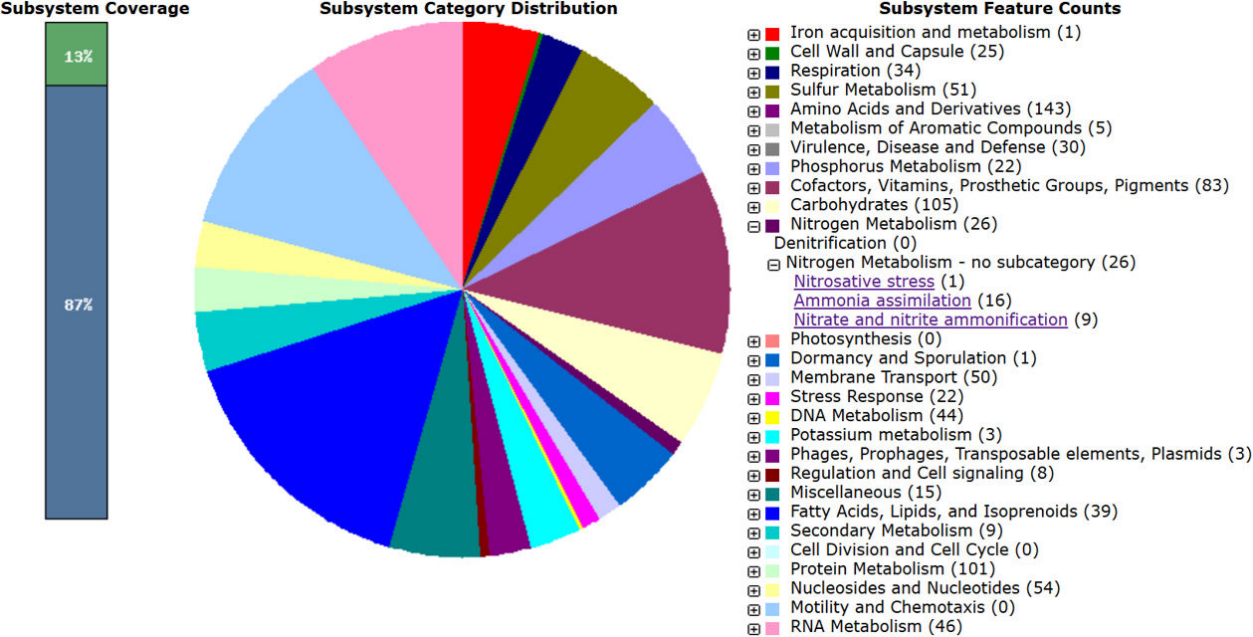
Subsystem category distribution of the draft genome of *Bremerella cremea* LHWP2 according to RAST annotation.

As analyzed using the RAST server with SEED viewer, the genome exhibits a guanine–cytosine content of 56.5%, encompasses 221 subsystems, encodes 5,304 protein-coding sequences, and contains 68 RNA genes, with an N50 value of 1,499,258 bp. Also, the results indicated that the most prominent functional categories in the genome were related to protein metabolism (101 genes), amino acids and derivatives (143 genes), and carbohydrates (105 genes), each containing over 100 genes (Fig. 1). Detailed subsystem analysis of this genome uncovered significant nitrogen metabolism pathways, which include mechanisms for nitrosative stress response (one gene), ammonia assimilation (16 genes), and nitrate and nitrite ammonification (nine genes). In addition to the RAST annotations, the gene call data were updated to reflect the PGAP statistics for the publicly available genome of strain LHWP2. According to the PGAP results, the genome contains 5,021 protein-coding sequences and 80 RNA genes.

The biological oxidation of ammonia (NH_3_) to nitrate (NO_3_^−^) via nitrite (NO_2_^−^) as part of the nitrogen cycle plays a pivotal role in engineered ecosystems, particularly in the removal of ammonium (NH_4_^+^) during the treatment of drinking water and wastewater. Therefore, the draft genome sequence of LHWP2 described in this report will contribute to understanding the nitrogen cycle on the southern coast of Korea.

## Data Availability

This whole genome project has been deposited in DDBJ/EMBL/GenBank under accession number JBGQOS000000000.1. Reads are available at the Sequence Read Archive (SRA) under accession number SRR30316889, BioProject number PRJNA1148086, and BioSample number SAMN43181681. RAST annotation files are available online at Figshare (https://doi.org/10.6084/m9.figshare.27600417.v1).
